# Invasion and Secondary Site Colonization as a Function of In Vitro Primary Tumor Matrix Stiffness: Breast to Bone Metastasis

**DOI:** 10.1002/adhm.202201898

**Published:** 2022-11-09

**Authors:** Lekha Shah, Ayşe Latif, Kaye J. Williams, Elena Mancuso, Annalisa Tirella

**Affiliations:** ^1^ Division of Pharmacy and Optometry Faculty of Biology Medicine and Health University of Manchester Oxford Road Manchester M13 9PL UK; ^2^ Nanotechnology and Integrated Bio‐Engineering Centre (NIBEC) Ulster University Shore Road Newtownabbey BT37 0QB UK; ^3^ BIOtech – Center for Biomedical Technologies Department of Industrial Engineering University of Trento Via delle Regole 101 Trento 38123 Italy; ^4^ Present address: BIOtech Center for Biomedical Technologies Department of Industrial Engineering University of Trento Via delle Regole 101 Trento 38123 Italy; ^5^ Present address: Engineering Ingegneria Informatica S.P.A. ‐ R&D Division Piazzale dell'Agricoltura 24 Rome 00144 Italy

**Keywords:** alginate hydrogels, breast‐to‐bone metastasis, decellularized PCL scaffolds, invasive potential, in vitro models, matrix stiffness

## Abstract

Increased breast tissue stiffness is correlated with breast cancer risk and invasive cancer progression. However, its role in promoting bone metastasis, a major cause of mortality, is not yet understood. It is previously identified that the composition and stiffness of alginate‐based hydrogels mimicking normal (1–2 kPa) and cancerous (6–10 kPa) breast tissue govern phenotype of breast cancer cells (including MDA‐MB‐231) in vitro. Here, to understand the causal effect of primary tumor stiffness on metastatic potential, a new breast‐to‐bone in vitro model is described. Together with alginate‐gelatin hydrogels to mimic breast tissue, 3D printed biohybrid poly‐caprolactone (PCL)‐composite scaffolds, decellularized following bone‐ECM deposition through Saos‐2 engraftment, are used to mimic the bone tissue. It is reported that higher hydrogel stiffness results in the increased migration and invasion capacity of MDA‐MB 231 cells. Interestingly, increased expression of osteolytic factors PTHrP and IL‐6 is observed when MDA‐MB‐231 cells pre‐conditioned in stiffer hydrogels (10 kPa, 3% w/v gelatin) colonize the bone/PCL scaffolds. The new breast‐to‐bone in vitro models herein described are designed with relevant tissue microenvironmental factors and could emerge as future non‐animal technological platforms for monitoring metastatic processes and therapeutic efficacy.

## Graphical abstract

This study delineates the importance of primary tumor stiffness in breast cancer invasion and bone metastasis using new in vitro models. Results suggests that stiffer hydrogels increase migration and invasive capability of MDA‐MB 231 cells. Moreover, preconditioning of cells in stiff breast‐mimicking hydrogel (10 kPa) is correlated with increased release of osteolytic factors while invading a bone‐like microenvironment.

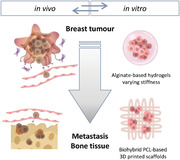

## 1 Introduction

Dynamic communication between cells and extracellular matrix (ECM) governs tissue structure and function. This homeostasis is deregulated in cancer wherein tumor cells and cancer associated fibroblasts actively remodel the ECM, leading to increased deposition, crosslinking and linearization of fibrillar components such as collagen.^[^ ^1^, ^2^ ^]^ Disrupted homeostasis leads to a change in biomechanical and biochemical nature of the ECM with modifications of matrix stiffness, tissue architecture and cell adhesion motifs.^[^ ^3^ ^]^ ECM modifications, and consequent tissue stiffening, are implicated in cell fate,^[^ ^4^ ^]^ cancer stemness,^[^ ^5^ ^]^ cancer progression,^[^ ^3^ ^]^ metastasis^[^ ^6^ ^]^ and therapeutic response in tumor tissue.^[^ ^7^, ^8^ ^]^ For example, tumor breast tissue is reported to be 3–6 times “stiffer” than the normal tissue,^[^ ^9^ ^]^ a characteristic shared across patients which, together with ECM changes, is commonly used as a clinical diagnostic technique with significance in tumor progression.^[^ ^10^ ^]^

Due to its importance in tumor diagnosis, progression and treatment, many studies have investigated effects of stiffness on breast cancer progression in vitro. For example, an increase in stiffness from 0.2 to 5 kPa in peptide crosslinked polyacrylamide gels was reported to perturb mammary acini formation by inducing unhindered growth and polarity loss in mammary epithelial cells.^[^ ^11^ ^]^ Stiff matrices were also observed to affect genome‐wide expression changes in nonmalignant breast epithelial cells and found to develop malignant phenotype by altering chromatin accessibility of genes.^[^ ^12^ ^]^ We recently reported the effect of alginate hydrogel stiffness on breast cancer stem cell (B‐CSC) populations, observing that high stiffness (6–10 kPa) together with acidic environment (pH 6.5) increase stem cell content (both epithelial and mesenchymal type of B‐CSCs) in MDA‐MB 231 and MCF‐7 breast cancer models.^[^ ^13^ ^]^ However, when it comes to correlating breast tissue stiffness with metastasis, very few studies have been designed to specifically illustrate how primary tumor stiffness and ECM direct invasion and metastasis to a secondary site (e.g., bone, lung, liver, brain). In fact, the vast majority of in vitro studies model the metastatic sites alone,^[^ ^14^, ^15^ ^]^ lacking the ability to understand the connection between primary and secondary (metastatic) site in the same model.

Bone is the most common site for breast cancer metastasis (≈60–70%) followed by lung, liver, and brain.^[^ ^16^ ^]^ What is known is that when a subpopulation of circulatory breast tumor cells encounters the bone microenvironment, the bone metastatic cycle starts. The initiator of the cycle is proposed to be the release of parathyroid hormone‐related protein (PTHrP) from tumor cells in response to the increased extracellular calcium^[^ ^17^ ^]^ and transforming growth factor‐*β* (TGF‐*β*) found in the bone.^[^ ^18^ ^]^ Tumor‐secreted cytokines such as interleukin‐6 (IL‐6)^[^ ^19^ ^]^ along with PTHrP^[^ ^20^ ^]^ instruct osteoblasts to release receptor activator of nuclear factor k*β* ligand (RANKL) which consequently stimulates osteoclast activity. This forms osteolytic bone lesions that further release trapped calcium and TGF‐*β* to induce cancer cell signaling, hence promoting the cycle.

The increase of tissue stiffness recorded in many solid tumors can condition cell phenotype, which is preserved even after the removal of such mechanical stimuli.^[^ ^21^, ^22^ ^]^ This is of particular relevance at later stages of tumor progression, i.e., invasion and metastasis, when cancer cells leave the primary tumor site and invade tissues with different ECM properties. Until now, the effect of primary tumor ECM on the conditioning of cells and their consequent response to the secondary site (such as bone) has not been deciphered. For this reason, 3D in vitro models, designed to link primary tumor and its metastatic site, offer a new opportunity to better understand the contribution of biomechanical and biochemical variations of the ECM on metastatic potential. In the present study, we have modeled for the first time on a single platform and in three dimension (3D), key properties of breast and bone tissue ECM, namely matrix composition, stiffness, density, porosity, and architecture to study breast‐to‐bone metastasis. We recently reported on the impact of normal (1–2 kPa) and tumor (>6 kPa) breast mimicking alginate–gelatin hydrogels on biomarker expression in MDA‐MB 231 cells.^[^ ^13^ ^]^ Based on our previous study, we used composite poly‐*ε*‐caprolactone (PCL) scaffolds containing inorganic compounds such as hydroxyapatite (HA), strontium HA (SrHA), and barium titanate (BaTiO_3_) to mimic the bone tissue stiffness (40–55 MPa) and porosity (35–45%).^[^ ^23^, ^24^ ^]^ Here, we have devised a new model combining breast tissue mimicking alginate hydrogels with bone mimicking PCL scaffolds. In particular, Saos‐2 cells (selected because of similar matrix deposition, cytokine, and growth factor pattern of primary osteoblasts^[^ ^25^, ^26^ ^]^) were cultured on composite PCL scaffolds allowing bone ECM deposition, following which decellularization procedures were optimized to retain deposited mineralized ECM and to increase the physiological relevance of bone‐mimicking scaffolds (named as biohybrid PCL scaffolds).

MDA‐MB 231 were preconditioned in alginate hydrogels of varying stiffness and the characterized for their cellular adhesion, migration, and 3D invasive potential. Then, alginate hydrogels and biohybrid PCL scaffolds were connected, and breast‐to‐bone metastasis was studied through in vitro assays. It was observed that migration, invasion, and osteolytic factor expression (PTHrP and IL‐6) in MDA‐MB 231 cells positively correlated with the increase of stiffness in hydrogels used to precondition these cells.

The proposed models are designed to recreate important properties of the breast tumor microenvironment in vitro, allowing the detection of cellular events directing biological processes (e.g., extravasation, ECM remodeling, colonization) toward bone metastatic sites. Refined models could be further used to better understand the complexity of metastatic cascade, with potential impact on the identification of new therapeutic modalities (e.g., mechanotherapeutics) and accelerate their translation to the clinic.

## 2 Experimental Section

### 2.1 General Cell Culture

Human breast adenocarcinoma cell line MDA‐MB 231 (HTB‐26, ATCC) and breast cancer bone homing cell line MDA‐IV (kindly provided by Prof I Holen, University of Sheffield, Sheffield, UK) were cultured in Dulbecco's modified Eagle medium (DMEM, D6546, Sigma‐Aldrich, UK) supplemented with 1% (v/v) L‐glutamine (G7513, Sigma‐Aldrich, UK), 10% (v/v) fetal bovine serum (FBS, F9665, Sigma‐Aldrich, UK) and 1% (v/v) penicillin‐streptomycin (P4333, Sigma‐Aldrich, UK). Human osteosarcoma cell line Saos‐2 (kindly provided by Dr O Tsikou, The University of Manchester, Manchester, UK) were cultured in McCoy's 5A media (M9309, Sigma‐Aldrich, UK) supplemented with 15% (v/v) FBS and 1% (v/v) Penicillin‐Streptomycin. All cell lines were tested negative for mycoplasma contamination by Mycoalert mycoplasma detection kit (LT07‐318, Lonza) prior use. Unless otherwise specified, all cell culture experiments were performed in a humidified 5% (v/v) CO_2_ air atmosphere at 37 °C in complete medium. Cells were used from passage 9 to 25.

### 2.2 3D Cell Culture: Encapsulation in Alginate–Gelatin Hydrogel Beads

#### 2.2.1 Preparation of Hydrogel Precursors and Crosslinking Solutions

Four different combinations of alginate and gelatin hydrogels (So‐L, So‐H, St‐L and St‐H) were selected, prepared and characterized as described in our previous study.^[^ ^13^ ^]^ Hydrogels match stiffness and composition of normal and tumor breast cancer tissue as summarized in **Table** **1**. Briefly, sodium alginic acid (G/M ratio of 0.7, Pro‐Alg, Chile) was dissolved in HEPES buffered saline (HBS) at a concentration of 3% and 6% (w/v). Obtained alginate solutions (aq.) were sterile filtered with 0.22 µm PES filter. Gelatin type A (G1890, Sigma‐Aldrich, UK) was dissolved in HBS, at a concentration of 2% and 6% (w/v). Gelatin solutions (aq.) were sterile filtered with 0.45 µm PVDF filter. Hydrogel precursor solutions were prepared by mixing (5 min, RT) different combination of alginate and gelatin solutions at selected concentrations with a 1:1 volume ratio (final hydrogel composition is reported in Table 1). Calcium chloride (CaCl_2_, C/1400/53, Fischer Scientific, UK) was dissolved in deionized water at concentrations of 100 × 10^−3^ m, 200 × 10^−3^ m and 300 × 10^−3^ m. Each solution (aq.) was sterile filtered with 0.22 µm PES filter prior use and stored at 4 °C.

**Table 1:** Composition of selected alginate (A)–gelatin (G) hydrogels varying compressive moduli (stiffness) and concentration of G (adhesion ligand content, hydrogel density). Sample ID are named as combination of properties: Soft (So, stiffness < 3 kPa), Stiff (St, stiffness > 6 kPa), low adhesion ligand (L, 1% w/v G), high adhesion ligand (H, 3% w/v G). Hydrogels were physically crosslinked using calcium chloride solutions (aq.)

Sample ID	Hydrogel composition		Adhesion ligand	Density^a)^	Stiffness^a)^	Pore size^a)^
	Polymer concentration [% w/v]	Crosslinking solution [×10^−3^ m]	Gelatin concentration		Compressive modulus [kPa]	[µm]
So‐L	A1.5G1	100	Low	2.5	1.8 ± 0.2	22.7 ± 6.2
So‐H	A1.5G3	100	High	4.5	2.4 ± 0.1	11.6 ± 2.2
St‐L	A3G1	300	Low	4	6.1 ± 0.2	17.1 ± 5.7
St‐H	A3G3	300	High	6	10.1 ± 0.5	12.1 ± 2.2

^a)^ Values are reported in ref. [13]. Of note, all hydrogels used in this study were tested before use to ensure quality and reproducibility of key properties (stiffness, pore size) across different batches.

#### 2.2.2 3D Breast Cancer Models: Alginate–Gelatin Hydrogels

MDA‐MB 231 cell pellets containing 2 × 10^6^ cells were resuspended in 1 mL of alginate‐gelatin solution (aq.) using the MICROMAN E viscous pipette (M1000E, Gilson, UK) and ensuring a homogeneous single cell suspension. The cell suspension was transferred in a sterile 1 mL syringe equipped with a 25G needle. A beaker was filled with sterile CaCl_2_ solution at known concentration (Table 1) and the cell suspension was ejected through the nozzle drop‐wise into the bath; the generated hydrogel beads were incubated in the CaCl_2_ solution (aq.) allowing gelation (10 min, RT). Spherical alginate‐gelatin hydrogel beads encapsulating cells were recovered using a cell strainer, washed twice in sterile HBS solution, immersed in complete cell culture media and finally transferred in the incubator.

### 2.3 Manufacturing of PCL‐Based Scaffolds

3D printed PCL‐based scaffolds (PCL, PCL/HA, PCL/BaTiO_3_ and PCL/SrHA) were manufactured as previously described^[^ ^23^, ^24^ ^]^ by using a 3D‐Bioplotter system (EnvisionTEC, Gladbeck; Germany). Briefly, the dispersion phase (10% w/w) of each composite formulation was mechanically mixed with the polymeric phase (RT). Subsequently, raw materials (4 g) in powder form were introduced into a stainless‐steel cartridge and processed as single extruded filament using a 22G nozzle, and according to the printing conditions reported in **Table** **2**. In order to increase pore interconnectivity, porous cylindrical (7 mm diameter) scaffolds were produced with a shifted architecture, made using a laydown pattern of 0/90° and an offset distance equal to half the distance between strands.

**Table 2:** Optimized printing parameters for 3D PCL‐based scaffolds

Sample ID	Temperature [°C]	Pressure [bar]	Speed [mm s^−1^]	Pre‐flow [s]	Post‐flow [s]
PCL	130	6	0.6	0.45	0.1
PCL/HA	130	6.5	0.6	0.75	0.1
PCL/BaTiO_3_	125	5.5	0.7	0.75	0.1
PCL/SrHA	130	6.2	0.6	0.75	0.1

#### 2.3.1 3D Bone Models: PCL‐Based Scaffolds

PCL‐based scaffolds were sterilized as described in our previous study.^[^ ^23^ ^]^ Sterile scaffolds were transferred to a 48 multi‐well (MW) plate, then 2 × 10^5^ Saos‐2 cells resuspended in complete media (50 µL) were gently pipetted on the top of each scaffold. Scaffolds were incubated allowing cell adhesion (30 min, 37 °C, 5% CO_2_), before addition of complete media (400 µL) in each well to cover the whole scaffold. After seven days of culture (37 °C, 5% CO_2_), the culture media was changed to osteoblast mineralization media (C‐27020, PromoCell, UK) to induce mineralization and changed thereafter every four days until the end point (i.e., day 28). Protocols for Alkaline phosphatase assay, Alizarin stain and ECM deposition on PCL scaffolds are reported in the Sections S1–S3 (Supporting Information).

#### 2.3.2 Decellularization of PCL‐Based Scaffolds: Biohybrid Bone Scaffolds

PCL‐based scaffolds were decellularized via a combination of mechanical and chemical methods,^[^ ^27^, ^28^ ^]^ performing all steps in sterile conditions. After 28 d of culture (end point), scaffolds were washed twice with sterile distilled water and frozen at ‐80 °C in water overnight. Scaffolds were thawed (2 h, RT), and the freeze‐thaw cycle was repeated twice to complete cell lysis. Following the mechanical decellularization step, scaffolds were washed twice with HBS and then incubated with sterile 1 mg mL^−1^ DNase (11284932001, Roche) solution diluted in HBS (1 h, 37 °C) to remove any residual nuclear debris. Further incubation with 0.05% (w/v) SDS sterile solution in HBS (15 min, RT) was performed to remove any remaining debris. Before any further cell culture experiments, scaffolds were washed (*n* = 3) with sterile HBS (5 min, RT) to remove any residual reagents. All solutions reported in this section were sterile filtered using 0.22 µm PES filter prior use.

### 2.4 Alginate–Gelatin Hydrogels and PCL‐Based Scaffolds: Breast‐to‐Bone Model

#### 2.4.1 Indirect Migration Model

**Figure 1:**
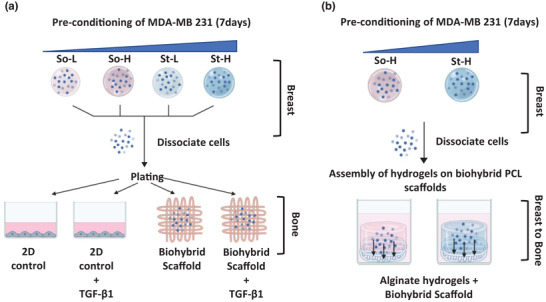
Schematic layout of the models used to study breast to bone metastasis: A) indirect migration and B) direct migration.

The indirect migration model was obtained by seeding preconditioned breast cancer cells onto the bone biohybrid PCL scaffolds (**Figure** **1**A). Prior to this step, MDA‐MB 231 cells were preconditioned in alginate‐gelatin hydrogels (Table 1) for 7 d, allowing adaptation to each distinctive breast tumor microenvironments (So‐L, So‐H, St‐L, and St‐H). After the pre‐conditioning step, MDA‐MB 231 cells were retrieved from alginate‐gelatin beads using the dissolution buffer.^[^ ^29^ ^]^ As controls, MDA‐MB 231 (nonconditioned) and MDA‐IV cells (nonconditioned) were cultured on standard tissue culture plastic (2D/TCP) wells, and detached with trypsin (T3924, Sigma‐Aldrich, UK) following standard protocols (3 min, 37 °C). All recovered cells were centrifuged at 600 g, resuspended in complete media, and then seeded directly onto the biohybrid PCL scaffold at a seeding density of 1×10^5^ per scaffold. As an additional control group, cells were also seeded on 2D/TCP wells at density of 2 × 10^4^ cells cm^−2^ (Figure 1A). For all the mentioned conditions, experiments were performed in complete cell culture media with/without addition of 5 ng mL^−1^ TGF‐*β*1 (100‐21, Peprotech, UK). Cells were cultured for 7 d in complete DMEM medium (37 °C, 5% CO_2_) for each condition, changing media every 2 d.

#### 2.4.2 Direct Migration Model

The direct migration model was designed to mimic the migration of breast cancer cells from primary (breast) to the secondary site (bone), i.e., from hydrogels to biohybrid PCL scaffolds (Figure 1B). Briefly, cells were retrieved from hydrogels (So‐H, St‐H) after 7 d using the dissociation buffer and re‐encapsulated in the same hydrogel type at a density of 2 × 10^6^ cells mL^−1^. A volume of cell‐hydrogel suspension (200 µL) was gently pipetted on top of the biohybrid PCL scaffold in a 48 MW plate and incubated (15 min, 4 °C) to allow physical gelation of gelatin and obtain cylindrical shaped cell‐laden hydrogels. Then CaCl_2_ (200 µL) was added to the well allowing crosslinking (10 min, RT).^[^ ^13^ ^]^ The combined scaffold was washed with HBS (*n* = 3), covered with complete DMEM and then incubated up to 7 d (37 °C, 5% CO_2_). Cell culture media was replaced every day.

### 2.5 Cell Proliferation Assay

Alamar blue assay (Deep Blue Cell Viability Kit, 424701, Biolegend) was used to analyze the proliferation of MDA‐MB 231 and MDA‐IV cells in biohybrid PCL scaffolds at days 1, 3, and 7.^[^ ^13^, ^23^ ^]^ Briefly, cell culture media was gently removed from each well, 400 µL of deep blue solution (10% v/v Deep blue viability reagent in complete cell culture media) was added to each well and incubated for 2 h (37 °C, 5% CO_2_). After incubation, solution (200 µL) was taken from each well, transferred to a 96‐ well plate, and immediately measured with Synergy‐2 (Biotek) plate reader (Ex 530–570 nm/Em 590–620 nm). The measurements were carried in triplicates (*n* = 3) for each experiment and values are plotted as mean ± standard deviation (SD) of *N* = 3 independent experiments.

### 2.6 Adhesion/Cell Spreading Assay

Eight‐well chambers slides (80826, Ibidi) were coated with 35 µg mL^−1^ (or 5 µg cm^−2^) collagen type I (sterile, 50201, Ibidi) diluted in acetic acid (17.5 × 10^−3^ m) and incubated for (1 h, RT), according to manufacturer's instructions. Collagen solution was slowly removed and chambers were washed with sterile PBS (*n* = 1). Similarly, fibronectin (F2006, Sigma) was diluted in sterile PBS and used at a concentration of 20 µg mL^−1^ (or 3 µg cm^−2^) (1 h, RT). After incubation, the fibronectin solution was removed and chambers were washed with sterile PBS (*n* = 1). After washing, both collagen and fibronectin‐coated chambers were left to air dry in sterile conditions (30 min, RT).

Cells preconditioned in hydrogels (So‐L, So‐H, St‐L, St‐H) for 7 d were recovered using the dissociation buffer and seeded on uncoated (control), collagen and fibronectin‐coated surfaces at a density of 1×10^4^ cells cm^−2^. After 45 min of incubation (37 °C, 5% CO_2_), cells were fixed with 4% (v/v) paraformaldehyde (PFA) solution and incubated (30 min, RT) with 1 µg mL^−1^ DAPI in PBS (D954, Sigma‐Aldrich, UK) and a 1:50 phalloidin Alexa‐568 in methanol (A12380, Invitrogen).

### 2.7 Scratch Assay

MDA‐MB 231 cells preconditioned in hydrogels (So‐L, So‐H, St‐L, St‐H) for 7 d were recovered using the dissociation buffer, seeded in six‐well plate at a density of 4 × 10^5^ cells per well and transferred to the incubator (37 °C, 5% CO_2_) allowing cell adhesion (24 h). A scratch was then performed in each well using a sterile 200 µL tip, then each well was washed with cell culture media to remove any cellular debris. Cells were supplemented with low serum media (1% v/v FBS in DMEM with 1% v/v L‐glutamine and 1% v/v PenStrep) to reduce cell proliferation,^[^ ^30^, ^31^ ^]^ and incubated up to 2 d (37 °C, 5% CO_2_).

### 2.8 Invasion Assay

Silicon inserts (80406, Ibidi) were cut in 4 × 4 mm pieces and placed at the center of each well (8‐well chambers, Ibidi) to create a cell free space (Figure S2A, Supporting Information). Collagen (50201, Ibidi) precursor mix was prepared following the supplier's instruction: collagen was diluted to a final concentration of 1 mg mL^−1^ in 10× DMEM (D2429, Sigma‐Aldrich) and sterile water, the pH was adjusted to 7.2–7.4 using sterile 1 m NaOH (aq.) (12963614, Fischer Scientific) and 7.5% (w/v) NaHCO_3_ solution (aq.) (S8761, Sigma‐Aldrich). MDA‐MB 231 cells were gently resuspended in the collagen pre‐gel solution at a density of 7.5 × 10^5^ cells mL^−1^. Immediately, cells‐collagen mix (200 µL) was pipetted outside the silicon insert and incubated allowing collagen gelation (30 min, 37 °C, 5% CO_2_). The central insert was removed and 1 mg mL^−1^ collagen solution (50 µL) was pipetted to fill the space with cell‐free collagen hydrogel. The whole setup was further incubated to complete gelation (20 min, 37 °C, 5% CO_2_).

After gelation, encapsulated cells were stained with Cytopainter red (ab138893, Abcam). Briefly, cells were incubated with 1× dye diluted in cell culture media for 1 h in the incubator and washed twice with 1× PBS followed by addition of cell culture media.

### 2.9 PThrP Expression

PThrP expression was detected from cell lysates at day 7 using the Human PTHrP Elisa kit (E‐EL‐H1478, Elabscience). MDA‐MB 231 cells were retrieved from PCL scaffold and well plates using trypsin (5 min, 37 °C), then recovered using fresh media. Cells were then centrifuged (3 min, 500 g), the supernatant removed and the pellet was incubated (5 min, 4 °C) in of lysis buffer (200 µL) composed of 1× RIPA buffer (ab156034, Abcam), complete mini protease inhibitor cocktail tablet (11836170001, Roche) and 1 × 10^−6^ m phenylmethylsulfonyl fluoride (PMSF). After 5 min on ice, the cell lysate was centrifuged (10 min, 4 °C, 2000 g) and supernatant was collected for further ELISA analysis. Sandwich ELISA was performed following manufacturer's instruction. Absorbance was measured at 450 nm using plate reader (Synergy‐2, Biotek). The blank OD values (lysate buffer only) were subtracted from absorbance of each sample, and the amount of PTHrP was determined from the standard calibration curve of known PTHrP concentration (pg mL^−1^).

BCA (bicinchoninic acid) protein assay (23228, Thermo Scientific) was used to measure the lysate concentration for each tested sample. Finally, PThrP concentration was normalized against its respective lysate concentration, and data were expressed as PTHrP pg mg^−1^ of protein values. The measurements were carried in duplicates (*n* = 2) for each experiment and values are plotted as mean ± SD of *N* = 3 independent experiments.

### 2.10 IL‐6 Release Quantification

To quantify IL‐6 release, fresh media was changed on day 6 and collected on day 7 (i.e., 24 h later), for each sample. Briefly, the collected medium was centrifuged (10 min, RT, 1000 g) and the supernatant was collected for further ELISA (human IL‐6 Kit, 550799, BD OptEIA) assay. The assay to detect IL‐6 release was performed according to supplier's instruction, with fresh complete media used as a blank. The amount of IL‐6 (pg mL^−1^) was determined from the standard calibration curve of known standard IL‐6 concentration. Each concentration was normalized against its respective cell proliferation data (Alamar data, Section 2.5) measured at day 7. The measurements were carried in duplicates (*n* = 2) for each experiment and values are plotted as mean ± SD of *N* = 3 independent experiments.

### 2.11 Image Acquisition and Analysis

Images were acquired using the fluorescent inverted microscope (Leica DMI6000, Leica Microsystems, UK) coupled with a 5.5 Neo sCMOS camera (Andor, UK), and equipped with: 2× objective (PLAN 2.5×/0.07, Leica), dry 10× objective (PL 10×/0.3 PH1, Leica), dry 20× objective (PL 20×/0.5 PH2, Leica), dry 63× objective (PL 63×/0.9 PH2, Leica), and filter cubes (A4, I3 and N2.1). The µManager software (v.1.46, Vale Lab, UCSF, USA) was used to control both microscope and camera, as well as to capture images.

#### 2.11.1 Adhesion Assay

Fluorescent images of cells were acquired using the 20× dry objective with filter cubes A4 (DAPI, nucleus) and N2.1 (Phalloidin Alexa‐568, F‐actin). Images were analyzed with ImageJ (v1.49p) for object identification and to measure the cell spread area. A minimum of 200 cells were analyzed per condition in *N* = 3 independent experiment, and individual cell area was calculated with ImageJ (v1.52a). The data is plotted as a dot plot and mean of individual cell area (*n* ≥ 200) for each condition.

#### 2.11.2 Scratch Assay

Brightfield images of the scratch were taken using the 10× dry objective at different time points (day 0, day 1, and day 2). The area of scratch invaded by cells was calculated using ImageJ (v1.52a) by measuring the combined cellular area in the scratch over time. The measurements were carried in duplicates (*n* = 2) for each condition and each time point, and the values are plotted as mean ± SD of *N* = 3 independent experiments.

#### 2.11.3 3D Collagen Invasion Assay

Fluorescent images of each well were acquired using the 2× dry objective and N2.1 filter cube, at each time point (day 0 and day 3). In order to measure invasion on the overall volume, z‐stacks (with z‐step of 50 µm) were acquired, and the maximum projection of each sample was obtained and analyzed with ImageJ (1.52a). For the analysis, thresholding of maximum projections and further conversion to binary, was used to count the total number of objects/cells in the acellular area for each condition. Images for analysis were acquired at day 0 and day 3. The measurements were carried in duplicates (*n* = 2) for each condition and the values are plotted as mean ± SD of *N* = 3 independent experiments.

### 2.12 Statistical Analysis

Significance for cell adhesion assay was analyzed by non‐parametric one‐way ANOVA with Dunn's post‐hoc multiple comparison test. Both migration and invasion assay were analyzed with one‐way analysis of variance (ANOVA) followed by Tukey's post‐hoc multiple comparison test. For PTHrP and IL‐6 analysis significance among conditions were analyzed by Two‐way ANOVA followed by Tukey's post‐hoc test. All significance tests and plotting of data were completed using GraphPad prism v9.1.0. *P*‐values were set at four different significance levels: *p ≤ 0.05, **p ≤ 0.01, ***p ≤ 0.001, ****p ≤ 0.0001.

## 3 Results and Discussion

### 3.1 Preconditioning of MDA‐MB 231 with High Stiffness: Effects on Migratory and Invasive Phenotype

MDA‐MB 231 are well known for their invasive potential and are commonly used as a model to study bone metastasis.^[^ ^32^, ^33^ ^]^ In this study, MDA‐MB 231 were selected and used to understand how breast tumor microenvironment (TME) properties (e.g., stiffness, density) could impact on various aspects of their metastatic potential. We examined the effect using four hydrogels, selected based on our previous study,^[^ ^13^ ^]^ on the invasive and migratory phenotypes of MDA‐MB 231 cells using three experiments: 1) adhesion ability (2D cell spread assay), 2) migration ability (2D scratch assay) and 3) invasion ability (3D collagen invasion assay). In all experiments, MDA‐MB 231 cells were allowed to adapt to the hydrogel “microenvironment” (Table 1) for 7 d prior to any investigation.

#### 3.1.1 Adhesion Ability

**Figure 2:**
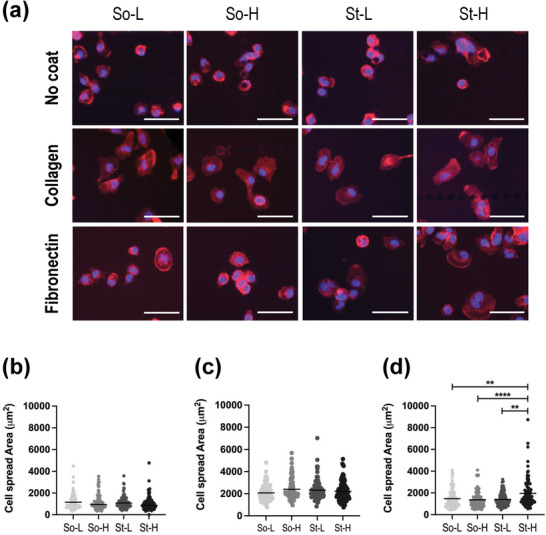
Cell spread area on: non‐coated, collagen and fibronectin covered plates. A) Immunofluorescent images of MDA‐MB 231 cells stained with DAPI (nuclei, blue) and phalloidin (F‐Actin, red) pre‐conditioned in the four alginate‐gelatin hydrogels and plated on non‐coated, collagen‐coated or fibronectin‐coated surfaces (Scale bars: 100 µm). Dot‐plot representations of individual cell area and mean of n ≥ 200 cells of: B) non‐coated, C), collagen‐coated, and D) and fibronectin‐coated. Significance was analyzed by non‐parametric one‐way ANOVA followed with Dunn's post‐hoc multiple comparison test. P‐values represented as *p ≤ 0.05, **p ≤ 0.01, ***p ≤ 0.001, ****p ≤ 0.0001.

The adhesion assay examined cellular adhesion and extent of membrane protrusion, both of which precede migratory and invasive phenotype. Preconditioned MDA‐MB 231 cells were left to adhere on non‐coated (2D/TCP), collagen and fibronectin coated plates (**Figure** **2**A–D). There was no significant variation in adhesion (i.e., number of cells that attached to the substrate) across all groups of pre‐conditioned cells and in all the substrates tested. Also, the individual cell spread area of all pre‐conditioned cells did not vary on noncoated and collagen‐coated plates. Interestingly, MDA‐MB‐231 cells preconditioned in St‐H hydrogels (high stiffness and density, high gelatin, and low pore size) showed increased cellular spreading in fibronectin‐coated plates (Figure 2A,D), illustrating the impact of TME stiffness and composition on cellular adhesion capacity against fibronectin adhesion motifs.

#### 3.1.2 Migration Ability

**Figure 3:**
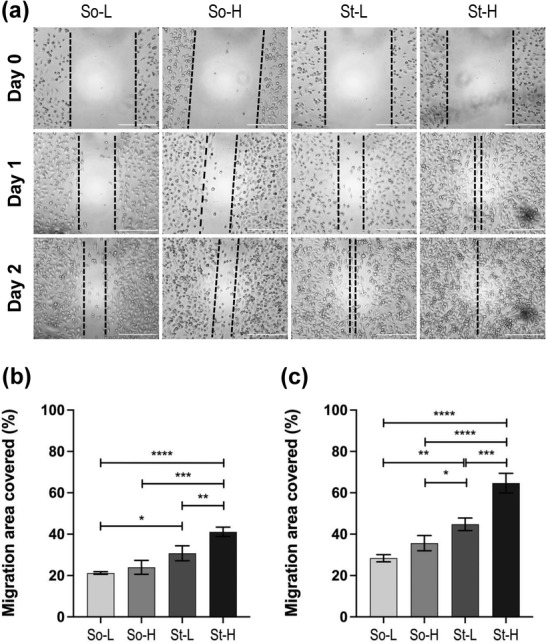
Scratch assay: A) Brightfield images of the scratch assay performed on cells pre‐conditioned in four hydrogels at day 0, day 1 and day 2 (Scale bars = 500 µm). Dashed black lines are used as visual representation and to help identifying cell migration over time in the different conditions tested. Scratch area (measured as µm^2^) covered by migratory cells is represented as % in comparison to blank area measured at day 0 for B) day 1 and C) day 2. The data is represented as mean ± SD of *n* = 2 wells, *N* = 3 independent experiments. Statistical significance was analyzed with one‐way analysis of variance (ANOVA) followed by Tukey's post‐hoc multiple comparison test. *P*‐values represented as *p ≤ 0.05, **p ≤ 0.01, ***p ≤ 0.001, ****p ≤ 0.0001.

Parallel to previous findings, MDA‐MB 231 cells preconditioned in stiff hydrogels (compressive modulus > 6 kPa) migrated faster than those preconditioned in softer hydrogels (compressive modulus < 3 kPa) as shown in (**Figure** **3**A–C). This was observed at both time points. Cells conditioned in So‐L versus St‐L hydrogels showed a 30% increase (*p* < 0.01) and So‐H versus St‐H showed 40% increase in covering the scratch‐wound area (*p* < 0.0001) at day 2 (Figure 3C). Within high stiffness hydrogels (St‐L, St‐H), cells cultured in St‐H showed increased migration capacity compared with St‐L (*p* ≤ 0.001, Figure 3C). Of note, there were no significant change in cell proliferation of these cells (Figure S1, Supporting Information). These results suggest the primary role of stiffness in this phenotype with gelatin content affecting cell migration capacity only when coupled with high stiffness.

#### 3.1.3 Invasion Ability

**Figure 4:**
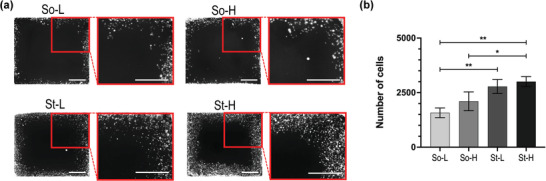
3D collagen invasion assay. A) Binary images of cytopainter red stained MDA‐MB 231 cells invading the acellular collagen hydrogel area at day 3, showing higher invasion of cells pre‐conditioned in St‐H hydrogels (Scale bars = 1000 µm). B) Number of invaded cells represented as mean ± SD of *n* = 2 and *N* = 3 independent experiments. Statistical significance was analyzed with one‐way analysis of variance (ANOVA) followed by Tukey's post‐hoc multiple comparison test. *P*‐values represented as *p ≤ 0.05, **p ≤ 0.01, ***p ≤ 0.001, ****p ≤ 0.0001).

Invasive potential in 3D was quantified by measuring the cellular ability to invade pristine collagen hydrogels. The invasion of MDA‐MB 231 cells preconditioned in hydrogels was tracked using cytopainter red staining. This stain is retained only by cells encapsulated in the hydrogel at day 0, excluding from the analysis any daughter cells whereby proliferation could confound measures of invasion (**Figure** **4**A, Figure S2B, Supporting Information). After 3 d, cells from stiffer hydrogels invaded collagen 1.5 times more than those conditioned in softer hydrogels (So‐L versus St‐L, *p* ≤ 0.01; So‐H vs St‐H, *p* ≤ 0.05) as shown in Figure 4B. Of note, no statistical difference was observed when comparing hydrogels with similar stiffness, varying gelatin content and density (So‐L vs So‐H, St‐L vs St‐H).

All these results suggest that preconditioning cells in TMEs of differing stiffness is critical to direct MDA‐MB 231 migratory and invasive phenotypes, whereas this correlation was not evidenced in differing gelatin concentrations. While this study only elucidates effects of gelatin‐related adhesion motifs, inclusion of other ECM components like fibronectin, laminin, and hyaluronic acid could better illustrate contributions of ECM composition on migration and invasion capacity.^[^ ^3^ ^]^

### 3.2 Biohybrid PCL Scaffolds: Decellularized PCL‐Based Scaffolds to Mimic Bone ECM

Circulatory breast cancer cells encounter a change in ECM properties when they extravasate to a bone microenvironment. In contrast to the collagenous matrix of breast tissue, the bone matrix consists of ≈60% inorganic matrix (majorly hydroxyapatite/HA) and ≈40% organic matrix (mainly collagen based).^[^ ^34^ ^]^ Highly vascular trabecular bone, which is a preferred site for bone metastasis,^[^ ^35^ ^]^ has high porosity (average ≈ 40%) and a heterogenous tissue stiffness that ranges from 4 to 80 MPa.^[^ ^36^ ^]^ Previous in vitro bone metastasis models use a collagenous matrix without taking into consideration the mechanical and ECM composition properties of the bone tissue.^[^ ^14^, ^15^ ^]^ For this reason, we used 3D‐printed composite PCL scaffolds with physiologically relevant mechanical properties (stiffness—40–55 MPa and porosity—35–45%) to mimic bone tissue.^[^ ^23^, ^24^ ^]^

**Figure 5:**
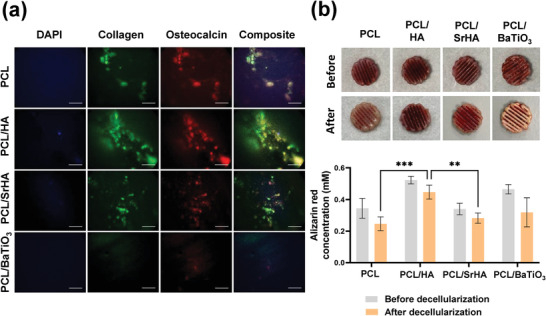
Selection of bone mimicking scaffold. Characterization of decellularized composite PCL scaffolds before/after decellularization of Saos‐2: A) Immunofluorescent images of nucleus (blue), collagen (green) and osteocalcin (red) stained PCL‐based scaffolds after decellularization steps (Scale bars: 50 µm). B) Images of PCL‐based scaffolds stained with Alizarin stain before and after decellularization and Alizarin stain concentration (× 10^−3^ m) from scaffolds before (gray) and after (orange) decellularization. Values are represented as mean ± SD of *N* = 3 independent experiments. Significance among conditions was analyzed by Two‐way ANOVA followed by Tukey's post‐hoc test. *P*‐values represented as *p ≤ 0.05, **p ≤ 0.01, ***p ≤ 0.001, ****p ≤ 0.0001.

PCL‐based scaffolds are known to retain ECM deposited by cells cultured on them, even after decellularization.^[^ ^27^, ^28^ ^]^ Hence, we compared four PCL‐based scaffolds (i.e., PCL, PCL/HA, PCL/SrHA, and PCL/BaTiO_3_^[^ ^23^, ^24^ ^]^) for their osteogenic potential, as well as ECM retaining ability after decellularization (**Figure** **5**). PCL‐composite scaffolds were cultured with Saos‐2 cells for up to 28 d, allowing deposition of bone‐ECM. Saos‐2 cells were selected as human osteoblast model because of their resemblance to primary osteoblasts in terms of similar matrix production, calcium deposition, and expression pattern of relevant cytokines and growth factors.^[^ ^25^, ^26^, ^37^, ^38^ ^]^ Osteoblast maturation was quantified in all PCL‐based scaffolds for up to 28 d using: cell proliferation, alkaline phosphatase (ALP) activity, calcium deposition (Alizarin stain) and ECM deposition (collagen and osteocalcin IF stain).^[^ ^39^ ^]^ Proliferation data suggests higher biocompatibility of composite PCL scaffolds (PCL/HA, PCL/SrHA, PCL/BaTiO_3_) when compared to pristine PCL scaffolds (Figure S3A, Supporting Information). PCL/BaTiO_3_ scaffolds were found to induce the highest amount of ALP in Saos‐2 cells (Figure S3B, Supporting Information); moreover, higher calcium deposition was found in both PCL/HA and PCL/BaTiO_3_ scaffolds (alizarin stain, Figure S4, Supporting Information). All PCL‐based scaffolds were positive for collagen and osteocalcin IF stain after 28 d of culture (Figure S5, Supporting Information).

Decellularization of biohybrid PCL scaffolds was further performed to assess: A) the capacity to retain deposited bone‐ECM, and B) the feasibility to remove cells from scaffolds under sterile conditions allowing their use in further studies (Figure 5). The decellularization steps were optimized for all PCL‐based scaffolds ( Figure S6, Supporting Information). The capability to retain deposited ECM was tested by examining the amount of collagen and osteocalcin (IF staining, Figure 5B), and calcium (Alizarin red, Figure 5C) in decellularized scaffolds. Pristine PCL, PCL/HA, and PCL/SrHA scaffolds retained both collagen and osteocalcin; whereas, PCL/BaTiO_3_ showed minimal ECM proteins with negligible staining detected (Figure 5A). Both PCL/HA and PCL/SrHA scaffolds were instead able to retain more calcium (about 10% reduction with respect to Saos‐2 colonized scaffolds); with PCL/HA scaffolds found to retain the highest amount of calcium (Figure 5B). Pristine PCL scaffolds and PCL/BaTiO_3_ scaffolds instead lost around 30% of previously quantified calcium deposition. Importantly, incubation with 1 mg mL^−1^ of DNase to lyse nuclear debris after mechanical disruption of cells was found critical to completely remove any residue from Saos‐2 cells (Figure S6, Supporting Information and Figure 5B). Based on these results, the PCL/HA scaffold was selected as the secondary bone metastatic scaffold for further invasion experiments, as it retained the highest amount of calcium and deposited ECM. This is subsequently referred to as biohybrid PCL/HA scaffold.

### 3.3 Indirect Migration of MDA‐MB 231 and MDA‐IV Cells in Biohybrid Scaffolds

**Figure 6:**
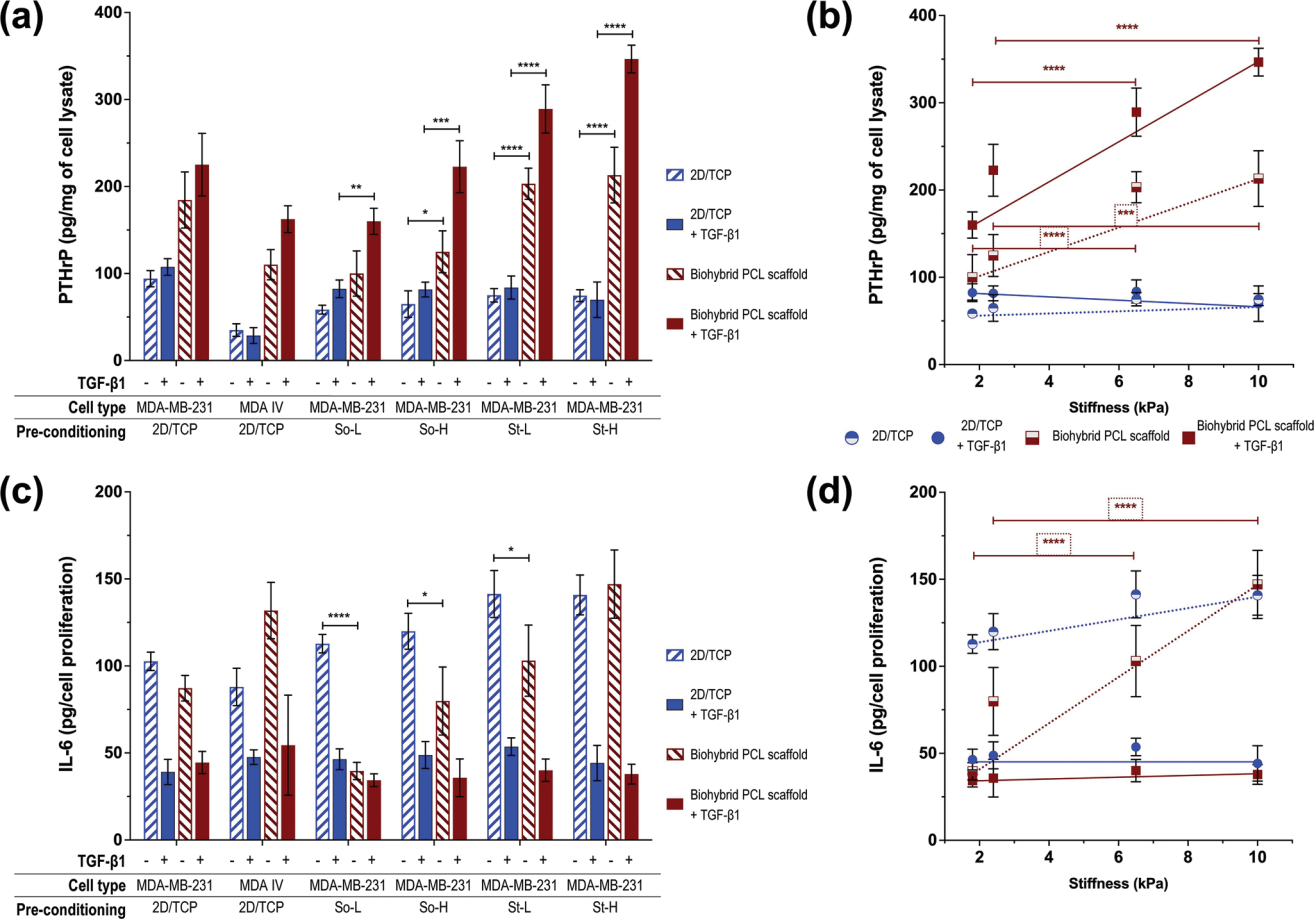
Indirect migration model: A) PTHrP expression and C) IL‐6 release quantified in MDA‐MB 231 and bone homing MDA‐IV cells plated on either 2D/TCP (blue) or biohybrid PCL/HA scaffolds (dark red) with and without 5 ng mL^−1^ TGF‐*β*1. Details of pre‐conditioning of MDA‐MB 231 cells is specified below each sample. Correlation plots are represented as a function of stiffness (kPa) of preconditioning hydrogels with: B) PTHrP expression and D) IL‐6 release. Dotted (‐TGF‐*β*1) and solid (+TGF‐*β*1) lines are used as guide for eyes (blue 2D/TCP; dark red biohybrid PCL/HA scaffolds). All values are represented as mean ± SD (*n* = 2 replicates, *N* = 3 independent experiments). Significance among conditions were analyzed by two‐way ANOVA followed by Tukey's post‐hoc test. *P*‐values represented as *p ≤ 0.05, **p ≤ 0.01, ***p ≤ 0.001, ****p ≤ 0.0001.

Based on the impact of the hydrogel properties on pre‐conditioning invasive and migratory potential of MDA‐MB 231 cells (Figures 2, 3, 4), two models were designed to study the impact of pre‐conditioning on breast‐to‐bone metastasis. The first model (i.e., indirect migration model) was designed to study the response of breast cancer cells to bone ECM, irrespective of their bone localizing potential (Figure 1A). In this, preconditioned MDA‐MB 231 cells were seeded onto biohybrid PCL/HA scaffold, and as a control, non‐conditioned MDA‐MB 231 and MDA‐IV (i.e., bone homing variant of MDA‐MB 231) were cultured on the same type of biohybrid PCL/HA scaffold. Expression of osteolytic factors (i.e., PTHrP, IL‐6) and cell proliferation was examined up to 7 d of culture. To further investigate the role of bone microenvironment growth factors released in response to increased osteoclast activity in bone metastasis, TGF‐*β*1 (5 ng mL^−1^) was supplemented as an additional variable of the model (**Figure** **6**).

#### 3.3.1 PTHrP Expression

At day 7, increased expression of PTHrP in MDA‐MB 231 cells plated on biohybrid PCL/HA scaffolds was measured as compared to those plated on controls (2D/TCP), confirming that exposure to bone‐specific ECM is essential in inducing PTHrP expression (Figure 6A). This is the first key step to trigger the bone metastasis cycle.^[^ ^17^ ^]^ Interestingly, a linear correlation between expression of PTHrP and stiffness of hydrogels (preconditioning step) was found when MDA‐MB 231 cells were cultured on biohybrid PCL/HA scaffolds. In particular, a twofold increase was observed when comparing stiffer conditioned groups to softer (*p* < 0.0001 for So‐L vs St‐L, *p* < 0.001 for So‐H vs St‐H) (Figure 6A,B). The same trend (proportional PTHrP expression with stiffness) was observed in the presence of TGF‐*β*1 (*p* < 0.0001 for So‐L vs St‐L and So‐H vs St‐H). Overall, the presence of TGF‐*β*1 increased PTHrP expression when cells were in biohybrid PCL/HA scaffolds (Figure 6B). Of note, no correlation of PTHrP expression with stiffness was observed when MDA‐MB 231 was cultured on TCP, regardless of the presence of TGF‐*β*1. The PTHrP expression pattern highlights the active role of both A) the primary site and cellular adaptation to the microenvironment (i.e., stiffness) and B) the composition and architecture of the secondary site (bone) in promoting osteolytic activity.

#### 3.3.2 IL‐6 Release

IL‐6 release increased twofold when cells were pre‐conditioned in high stiffness hydrogels and seeded on the biohybrid PCL/HA scaffolds, with no variation recorded in TCP controls (Figure 6C,D). Surprisingly, TGF‐*β*1 supplementation caused reduction of IL‐6 release in all the tested conditions, with no differences between conditioned and non‐conditioned cells (Figure 6C,D).

#### 3.3.3 Proliferation

Regarding MDA‐MB 231 proliferation (day 3, day 7) in biohybrid PCL/HA scaffolds, no significant difference was observed between conditioned and nonconditioned cells (Figure S7A, Supporting Information). However, the presence of TGF‐*β*1 induced an increase in proliferation, found proportional with hydrogel stiffness used to pre‐condition the cells. MDA‐IV cell proliferation was found similar to MDA‐MB 231 pre‐conditioned in St‐H hydrogels (Figure S7B, Supporting Information).

In summary, MDA‐MB 231 cells express more PTHrP and exhibit proportional increase in response to stiffness of the conditioning TME (primary tumor), only when cultured in bone‐mimicking scaffolds (biohybrid PCL/HA scaffold, secondary site). IL‐6 release is also positively correlated to increasing stiffness of the preconditioning hydrogel. Results support the need of models better representing the characteristics of tissues of interest, (i.e., 3D bioactive scaffolds) rather than conventional models (i.e., 2D/TCP surfaces).

The inclusion of TGF‐*β*1 in the model did impact on both PTHrP and IL‐6 release, and aligns with previous studies. A clear relationship of increased PTHrP expression induced by TGF‐*β* in breast‐to‐bone metastasis is reported;^[^ ^18^, ^40^ ^]^ and no correlation with IL‐6 expression is found in the same context. Studies conducted in other model systems report complex and mixed crosstalk between TGF‐*β* and IL‐6. In intestinal epithelial cells, TGF‐*β* dampens IL‐6 signaling but reports no direct effect on its expression.^[^ ^41^ ^]^ In biliary tract cancer, they work synergistically to induce EMT and chemotherapy resistance.^[^ ^42^ ^]^ Both TGF‐*β* ^[^ ^43^ ^]^ and IL‐6^[^ ^44^ ^]^ have pleiotropic effects which make their interaction complex, hence further investigation is needed to draw solid conclusions about their interaction within this model.

Based on the presented results, it is possible to conclude that the indirect migration model mimics the initial steps of bone metastatic cycle, and in particular the release of osteolytic factors by cancer cells in response to the bone ECM. As colonization is a later stage of the metastatic cycle and relies on complex interactions with other components of the microenvironment (e.g., osteoblasts, osteoclasts, vascularization), further in‐depth understanding of metastatic onsets and clinical observations could be achieved by including additional cues in the model.

### 3.4 Direct Migration of MDA‐MB 231 and MDA‐IV from Hydrogels to Biohybrid Scaffolds

Direct migration model was designed to investigate migration/localization potential of MDA‐MB‐231 and MDA‐IV to biohybrid PCL scaffolds (i.e., bone, secondary site), with alginate hydrogels encapsulating cells placed directly on the biohybrid PCL scaffolds to allow for breast cancer cell migration (Figure 1B). Two hydrogels varying only in stiffness were selected (So‐H and St‐H) based on the results of the indirect migration model (Figure 6).

#### 3.4.1 Migration

To assess migration using the direct migration model, the number of viable cells migrated into biohybrid PCL scaffolds was determined after 7 d (endpoint) using the deep blue viability assay. We found that MDA‐MB 231 and bone homing MDA‐IV cells migrate with similar rate (no statistical difference between cell type), with a higher migration observed from softer hydrogel than stiff hydrogel (So‐H > St‐H, Figure S8, Supporting Information). Although it was observed that pre‐conditioning in stiffer hydrogels correlates with higher migration rate in collagen hydrogels (Figure 4), we found that cells within So‐H hydrogel migrate faster towards the biohybrid PCL scaffolds than the ones in St‐H hydrogel. Here, hydrogel pore size and composition (Table 1) could be key players in directing cells migration from one microenvironment (hydrogel) to another (biohybrid PCL scaffolds), based on the hypothesis that smaller pore size can slow cell migration.^[^ ^45^ ^]^ Results reflect what is reported in literature, with invasion of cancer cells being predominant within softer and less dense alginate hydrogels,^[^ ^46^ ^]^ but showcasing increased invasion in collagen hydrogels when pre‐conditioned with higher stiffness first.^[^ ^22^ ^]^

#### 3.4.2 PTHrP and IL‐6 Expression

**Figure 7:**
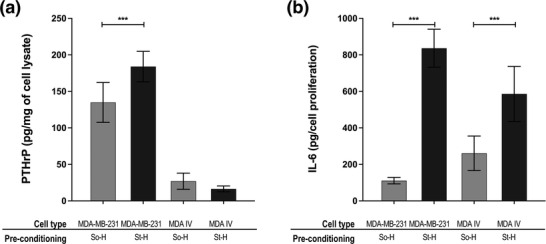
Direct migration model: A) PTHrP expression and B) IL‐6 release in MDA‐MB 231 and MDA‐IV cells preconditioned in So‐H (gray) and St‐H (black). Of note, results consider only cells that have migrated into the biohybrid PCL/HA scaffolds. All values are represented as mean ± SD (*n* = 2 replicates, *N* = 3 independent experiments). Significance among conditions were analyzed by two‐way ANOVA followed by Tukey's post‐hoc test. *P*‐values represented as *p ≤ 0.05, **p ≤ 0.01, ***p ≤ 0.001, ****p ≤ 0.0001.

Interestingly, PTHrP expression in MDA‐MB 231 cells pre‐conditioned in St‐H hydrogels and migrated to biohybrid PCL scaffolds was found 1.4‐fold higher than So‐H hydrogels (*p* < 0.05) (**Figure** **7**A). Similarly, IL‐6 release was measured to be eightfold higher in migrated cells pre‐conditioned in stiffer hydrogels (St‐H ves So‐H, *p* < 0.0001) (Figure 7B). This suggests that while less cells migrated from St‐H hydrogels, these have higher expression of osteolytic factors that could lead to higher metastatic load. These results support data reported by Watson et al. who performed functional osteolytic assay using mouse models. Authors reported progressive osteolysis and increased osteolytic lesions when cells preconditioned in higher stiffness (8 kPa) were injected (intraventricular injection) in mouse models compared to cells pre‐conditioned in low stiffness (0.5 kPa).^[^ ^22^ ^]^

Results from the direct migration model suggest that high primary tumor stiffness is linked with increased osteolysis. Although only a snapshot of the microenvironment, our results suggest that the expression of osteolytic factors could be a better indicator of bone metastatic load than cell migration/motility.

### 3.5 Response of MDA‐IV Cells in Direct and Indirect Migration Model

#### 3.5.1 PTHrP Expression

We observed that intrinsic PTHrP expression is lower in MDA‐IV cells compared to parental MDA‐MB 231 cell line when cultured in 2D/TCP (Figure 6A). Interestingly, when MDA‐IV cells are cultured in biohybrid PCL scaffolds, PTHrP expression increases regardless of TGF‐*β*1 supplementation, in line with MDA‐MB 231 (Figure 6A). This confirms that 3D bone microenvironment induces expression of osteolytic factors also in MDA‐IV cells. Surprisingly, the expression of PTHrP in MDA‐IV was low in the direct migration model and did not vary as a function of stiffness of the pre‐conditioning environment (Figure 7A). As MDA‐IV cells exclusively metastasize to bone in vivo,^[^ ^47^ ^]^ these results could suggest that while PTHrP is an important factor in bone remodeling and colonization, it might not be as important to localize/migrate to the bone tissue. This is confirmed clinically where studies on patient tumors indicate that positive PTHrP expression in primary tumor is in fact linked to lower bone metastasis.^[^ ^48^, ^49^, ^50^ ^]^

#### 3.5.2 IL‐6 Release

No difference was found in IL‐6 release between MDA‐IV and MDA‐MB‐231 cells when cultured in 2D/TCP. However, the indirect migration models showcased that levels of IL‐6 increased in MDA‐IV when cultured in biohybrid PCL scaffolds, aligning only with MDA‐MB 231 pre‐conditioned in the stiffer microenvironment (St‐H, Figure 6C). Similarly, MDA‐IV cultured in the direct migration models released IL‐6 proportionally to the preconditioning stiffness (twofold increase in cells cultured in stiff hydrogels vs soft, Figure 7B). These results suggest that IL‐6 might be a bone metastatic marker for both localization and remodeling. The importance of IL‐6 cytokine in the initiation of breast cancer invasion and metastasis^[^ ^51^, ^52^ ^]^ as well as its involvement in bone metastasis and osteolytic activity has been documented^[^ ^19^, ^53^ ^]^ in animal models, which aligns with our findings for IL‐6.

In summary, results obtained with MDA‐IV in both direct and indirect migration models suggest that it is possible to de‐couple two different properties of breast‐to‐bone metastasis namely, localization and osteolytic/remodeling potential, while also examining the importance of different markers in these processes.

Noteworthy, many studies have engineered breast to bone metastasis in vitro.^[^ ^14^, ^15^, ^54^, ^55^, ^56^ ^]^ However, these model the extravasation alone and typically use collagen hydrogels, known to poorly represent the bone tissue architecture.^[^ ^14^, ^15^ ^]^ Other studies use directly ex vivo bone tissue from donors, which have inherent variability and limited availability due to the nature of its sourcing.^[^ ^54^, ^55^ ^]^ The in vitro models described in this study not only pave the way for better integration of primary tumor and secondary site, it also includes relevant ECM properties modeled with the use of standardized and reproducible biomaterials.

## 4 Conclusions

Interactions between ECM and cancer cells direct phenotypes and matrix remodeling during tumor progression. In this study, engineering such matrix changes in vitro was found to be useful in isolating specific pattern of ECM properties and understanding their impact on biological processes. We used specific 3D in vitro models to elucidate effects of primary tumor matrix stiffness and gelatin‐related adhesion motifs on various dimensions of breast cancer metastasis (i.e., adhesion, migration, 3D invasion, 3D secondary metastatic site response). Stiffer primary tumor microenvironments (compressive moduli > 6 kPa) were found to be essential in inducing migratory and invasive phenotypes in MDA‐MB 231 cells.

In recreating aspects of breast‐to‐bone metastasis in vitro, we combined a 3D model matching human breast cancer tissue properties with a 3D model matching the properties of bone ECM. Two models were set‐up to study metastatic onsets from breast‐to‐bone and focused on bone localization versus bone remodeling. The indirect migration model evidenced that both primary tumor stiffness and secondary site ECM composition are essential in regulating expression of osteolytic factors PTHrP and IL‐6, and hence could affect osteolytic bone remodeling. The direct migration model instead evidenced that high stiffness in the breast tumor is linked to high IL‐6 activity, an important factor for metastasis initiation as well as osteolysis.

The proposed engineered in vitro models, in accordance with previous in vivo studies, were able to confirm that high primary tumor stiffness is linked to increased migration, invasion, and osteolytic factor expression in breast cancer cells. Moreover, the models allowed decoupling of different ECM properties to elucidate effects of both primary tumor ECM and secondary site ECM in breast‐to‐bone metastasis. Further inclusion of primary/patient‐derived cells of known metastatic status in the proposed engineered in vitro models would be useful to predict the possibility of bone metastasis and used as a platform to test therapeutic efficacy or to tailor personalized treatments.

## Conflict of Interest

The authors declare no conflict of interest.

## Supporting information

Supporting Information

## Data Availability

The data that support the findings of this study are available from the corresponding author upon reasonable request.

## References

[1] T. R. Cox , J. T. Erler , Dis. Models Mech. 2011, 4, 165. 10.1242/dmm.004077 PMC3046088 21324931

[2] A. E. Yuzhalin , S. Y. Lim , A. G. Kutikhin , A. N. Gordon‐Weeks , Biochim. Biophys. Acta, Rev. Cancer 2018, 1870, 207. 30316942 10.1016/j.bbcan.2018.09.002

[3] J. Winkler , A. Abisoye‐Ogunniyan , K. J. Metcalf , Z. Werb , Nat. Commun. 2020, 11, 5120. 33037194 10.1038/s41467-020-18794-x PMC7547708

[4] J. I. Lopez , J. K. Mouw , V. M. Weaver , Oncogene 2008, 27, 6981. 19029939 10.1038/onc.2008.348 PMC2648514

[5] S. Nallanthighal , J. P. Heiserman , D. J. Cheon , Front. Cell Dev. Biol. 2019, 7, 86. 31334229 10.3389/fcell.2019.00086 PMC6624409

[6] J. S. Di Martino , T. Akhter , J. J. Bravo‐Cordero , Cancers 2021, 13, 4916. 34638400 10.3390/cancers13194916 PMC8507703

[7] H. Denys , G. Braems , K. Lambein , P. Pauwels , A. Hendrix , A. De Boeck , V. Mathieu , M. Bracke , O. De Wever , Curr. Pharm. Des. 2009, 15, 1373. 19355975 10.2174/138161209787846711

[8] E. Henke , R. Nandigama , S. Ergün , Front. Mol. Biosci. 2020, 6, 160. 32118030 10.3389/fmolb.2019.00160 PMC7025524

[9] A. Samani , J. Zubovits , D. Plewes , Phys. Med. Biol. 2007, 52, 1565. 17327649 10.1088/0031-9155/52/6/002

[10] S. X. Zhang , L. Liu , W. Zhao , Trends Cancer 2018, 4, 268. 29606309 10.1016/j.trecan.2018.02.006 PMC6226275

[11] M. J. Paszek , N. Zahir , K. R. Johnson , J. N. Lakins , G. I. Rozenberg , A. Gefen , C. A. Reinhart‐King , S. S. Margulies , M. Dembo , D. Boettiger , D. A. Hammer , V. M. Weaver , Cancer Cells 2005, 8, 241. 10.1016/j.ccr.2005.08.010 16169468

[12] R. S. Stowers , A. Shcherbina , J. Israeli , J. J. Gruber , J. Chang , S. Nam , A. Rabiee , M. N. Teruel , M. P. Snyder , A. Kundaje , O. Chaudhuri , Nat. Biomed. Eng. 2019, 3, 1009. 31285581 10.1038/s41551-019-0420-5 PMC6899165

[13] L. Shah , A. Latif , K. J. Williams , A. Tirella , Acta Biomater. 2022, 152, 273. 36087866 10.1016/j.actbio.2022.08.074

[14] S. Bersini , J. S. Jeon , G. Dubini , C. Arrigoni , S. Chung , J. L. Charest , M. Moretti , R. D. Kamm , Biomaterials 2014, 35, 2454. 24388382 10.1016/j.biomaterials.2013.11.050 PMC3905838

[15] J. S. Jeon , S. Bersini , M. Gilardi , G. Dubini , J. L. Charest , M. Moretti , R. D. Kamm , Proc. Natl. Acad. Sci. USA 2015, 112, 214. 25524628 10.1073/pnas.1417115112 PMC4291627

[16] M. T. Chen , H. F. Sun , Y. Zhao , W. Y. Fu , L. P. Yang , S. P. Gao , L. D. Li , H. L. Jiang , W. Jin , Sci. Rep. 2017, 7, 9254. 28835702 10.1038/s41598-017-10166-8 PMC5569011

[17] J. L. Sanders , N. Chattopadhyay , O. Kifor , T. Yamaguchi , R. R. Butters , E. M. Brown , Endocrinology 2000, 141, 4357. 11108243 10.1210/endo.141.12.7849

[18] J. J. Yin , K. Selander , J. M. Chirgwin , M. Dallas , B. G. Grubbs , R. Wieser , J. Massagué , G. R. Mundy , T. A. Guise , J. Clin. Invest. 1999, 103, 197. 9916131 10.1172/JCI3523 PMC407876

[19] P. Palmqvist , E. Persson , H. H. Conaway , U. H. Lerner , J. Immunol. 2002, 169, 3353. 12218157 10.4049/jimmunol.169.6.3353

[20] R. J. Thomas , T. A. Guise , J. J. Yin , J. Elliott , N. J. Horwood , T. J. Martin , M. T. Gillespie , Endocrinology 1999, 140, 4451. 10499498 10.1210/endo.140.10.7037

[21] B. F. Matte , A. Kumar , J. K. Placone , V. G. Zanella , M. D. Martins , A. J. Engler , M. L. Lamers , J. Cell Sci. 2019, 132, 224360. 10.1242/jcs.224360 PMC6340137 30559248

[22] A. W. Watson , A. D. Grant , S. S. Parker , S. Hill , M. B. Whalen , J. Chakrabarti , M. W. Harman , M. R. Roman , B. L. Forte , C. C. Gowan , R. Castro‐Portuguez , L. K. Stolze , C. Franck , D. A. Cusanovich , Y. Zavros , M. Padi , C. E. Romanoski , G. Mouneimne , Cell Rep. 2021, 35, 109293. 34192535 10.1016/j.celrep.2021.109293 PMC8312405

[23] E. Mancuso , L. Shah , S. Jindal , C. Serenelli , Z. M. Tsikriteas , H. Khanbareh , A. Tirella , Mater. Sci. Eng., C 2021, 126, 112192. 10.1016/j.msec.2021.112192 34082989

[24] D. Pierantozzi , A. Scalzone , S. Jindal , L. Stīpniece , K. Šalma‐Ancāne , K. Dalgarno , P. Gentile , E. Mancuso , Compos. Sci. Technol. 2020, 191, 108069.

[25] G. Bilbe , E. Roberts , M. Birch , D. B. Evans , Bone 1996, 19, 437. 8922641 10.1016/s8756-3282(96)00254-2

[26] E. M. Czekanska , M. J. Stoddart , R. G. Richards , J. S. Hayes , Eur. Cells Mater. 2012, 24, 1. 10.22203/ecm.v024a01 22777949

[27] B. A. Dikici , G. C. Reilly , F. Claeyssens , ACS Appl. Mater. Interfaces 2020, 12, 12510. 32100541 10.1021/acsami.9b23100 PMC7146758

[28] B. Nayak , G. M. Balachander , S. Manjunath , A. Rangarajan , K. Chatterjee , Colloids Surf., B 2019, 180, 334. 10.1016/j.colsurfb.2019.04.056 31075687

[29] J. M. R. de la Rosa , J. Wubetu , N. Tirelli , A. Tirella , Biomed. Phys. Eng. Express 2018, 4, 045010. 10.1088/2057-1976/aac1c9 37596738

[30] L. G. Rodriguez , X. Wu , J. L. Guan , Methods Mol. Biol. 2005, 294, 23. 15576902 10.1385/1-59259-860-9:023

[31] B. R. B. Pires , A. L. Mencalha , G. M. Ferreira , W. F. De Souza , J. A. Morgado‐Díaz , A. M. Maia , S. Corrêa , E. S. F. W. Abdelhay , PLoS One 2017, 12, e0169622. 28107418 10.1371/journal.pone.0169622 PMC5249109

[32] X. Dai , H. Cheng , Z. Bai , J. Li , J. Cancer 2017, 8, 3131. 29158785 10.7150/jca.18457 PMC5665029

[33] A. Bellahcène , R. Bachelier , C. Detry , R. Lidereau , P. Clézardin , V. Castronovo , Breast Cancer Res. Treat. 2006, 101, 135. 17028989 10.1007/s10549-006-9279-8

[34] X. Lin , S. Patil , Y. G. Gao , A. Qian , Front. Pharmacol. 2020, 11, 757. 32528290 10.3389/fphar.2020.00757 PMC7264100

[35] J. Fornetti , A. L. Welm , S. A. Stewart , J. Bone Miner. Res. 2018, 33, 2099. 30476357 10.1002/jbmr.3618

[36] A. Syahrom , M. R. Abdul Kadir , J. Abdullah , A. Öchsner , Med. Biol. Eng. Comput. 2011, 49, 1393. 21947767 10.1007/s11517-011-0833-0

[37] D. J. McQuillan , M. D. Richardson , J. F. Bateman , Bone 1995, 16, 415. 7605701 10.1016/8756-3282(95)90186-8

[38] M. Prideaux , A. R. Wijenayaka , D. D. Kumarasinghe , R. T. Ormsby , A. Evdokiou , D. M. Findlay , G. J. Atkins , Calcif. Tissue Int. 2014, 95, 183. 24916279 10.1007/s00223-014-9879-y

[39] A. Rutkovskiy , K.‐O. Stensløkken , I. J. Vaage , Med. Sci. Monit. Basic Res. 2016, 22, 95. 27667570 10.12659/MSMBR.901142 PMC5040224

[40] A. Chiechi , D. L. Waning , K. R. Stayrook , J. T. Buijs , T. A. Guise , K. S. Mohammad , Adv. Biosci. Biotechnol. 2013, 4, 15. 24558636 10.4236/abb.2013.410A4003 PMC3928102

[41] B. Walia , L. Wang , D. Merlin , S. V. Sitaraman , FASEB J. 2003, 17, 2130. 14500551 10.1096/fj.02-1211fje

[42] D. Yamada , S. Kobayashi , H. Wada , K. Kawamoto , S. Marubashi , H. Eguchi , H. Ishii , H. Nagano , Y. Doki , M. Mori , Eur. J. Cancer 2013, 49, 1725. 23298711 10.1016/j.ejca.2012.12.002

[43] M. Veldhoen , B. Stockinger , Trends Immunol. 2006, 27, 358. 16793343 10.1016/j.it.2006.06.001

[44] T. Kishimoto , Arthritis Res. Ther. 2006, 8, S2. 10.1186/ar1916 PMC3226075 16899106

[45] J. Tien , U. Ghani , Y. W. Dance , A. J. Seibel , M. Ç. Karakan , K. L. Ekinci , C. M. Nelson , iScience 2020, 23, 101673. 33163933 10.1016/j.isci.2020.101673 PMC7599434

[46] M. P. Pebworth , S. A. Cismas , P. Asuri , PLoS One 2014, 9, e110453. 25310593 10.1371/journal.pone.0110453 PMC4195729

[47] F. Nutter , I. Holen , H. K. Brown , S. S. Cross , C. Alyson Evans , M. Walker , R. E. Coleman , J. A. Westbrook , P. J. Selby , J. E. Brown , P. D. Ottewell , Endocr.‐Relat. Cancer 2014, 21, 327. 24413608 10.1530/ERC-13-0158

[48] M. A. Henderson , J. A. Danks , J. M. Moseley , J. L. Slavin , T. L. Harris , M. R. Mckinlay , J. L. Hopper , T. J. Martin , M. A. Henderson , T. L. Harris , M. R. Mckinlay , J. A. Danks , J. M. Moseley , T. J. Martin , S. Vincent’ , JNCI, J. Natl. Cancer Inst. 2001, 93, 234. 11158193 10.1093/jnci/93.3.234

[49] M. A. Henderson , J. A. Danks , J. L. Slavin , G. B. Byrnes , P. F. M. Choong , J. B. Spillane , J. L. Hopper , T. J. Martin , Cancer Res. 2006, 66, 2250. 16489028 10.1158/0008-5472.CAN-05-2814

[50] C. M. Edwards , R. W. Johnson , Front. Oncol. 2021, 11, 644303. 33828987 10.3389/fonc.2021.644303 PMC8019909

[51] C. Dethlefsen , G. Højfeldt , P. Hojman , Breast Cancer Res. Treat. 2013, 138, 657. 23532539 10.1007/s10549-013-2488-z

[52] H. Jayatilaka , J. M. Phillip , Breast Cancer Manage. 2019, 8, BMT20.

[53] T. Ara , Y. A. DeClerck , Eur. J. Cancer 2010, 46, 1223. 20335016 10.1016/j.ejca.2010.02.026 PMC2917917

[54] A. Marturano‐Kruik , M. M. Nava , K. Yeager , A. Chramiec , L. Hao , S. Robinson , E. Guo , M. T. Raimondi , G. Vunjak‐Novakovic , Proc. Natl. Acad. Sci. USA 2018, 115, 1256. 29363599 10.1073/pnas.1714282115 PMC5819403

[55] I. Holen , F. Nutter , J. M. Wilkinson , C. A. Evans , P. Avgoustou , P. D. Ottewell , Clin. Exp. Metastasis 2015, 32, 689. 26231669 10.1007/s10585-015-9737-y

[56] A. Tirella , in Microfluidics and Multi Organs on Chip (Ed: P. V. Mohanan ) Springer, Berlin 2022, p. 681.

