# Supplementary material for: Invasion and Secondary Site Colonization as a Function of In Vitro Primary Tumor Matrix Stiffness: Breast to Bone Metastasis
Source: Adv Healthc Mater. 2022 Nov 9;12(3):2201898. doi: 10.1002/adhm.202201898 (PMC11468571; doi:10.1002/adhm.202201898)
Supplement: Supplementary file 1 — Supporting Information [file ADHM-12-2201898-s001.pdf]

ADVANCED HEALTHCARE MATERIALS

Supporting Information

for *Adv. Healthcare Mater.*, DOI 10.1002/adhm.202201898

Invasion and Secondary Site Colonization as a Function of In Vitro Primary Tumor Matrix Stiffness: Breast to Bone Metastasis

*Lekha Shah, Ayşe Latif, Kaye J. Williams, Elena Mancuso and Annalisa Tirella**

Supporting Information

Invasion and secondary site colonization as a function of in vitro primary tumor matrix stiffness: breast to bone metastasis

*Lekha Shah, Ayşe Latif, Kaye J. Williams, Elena Mancuso and Annalisa Tirella**

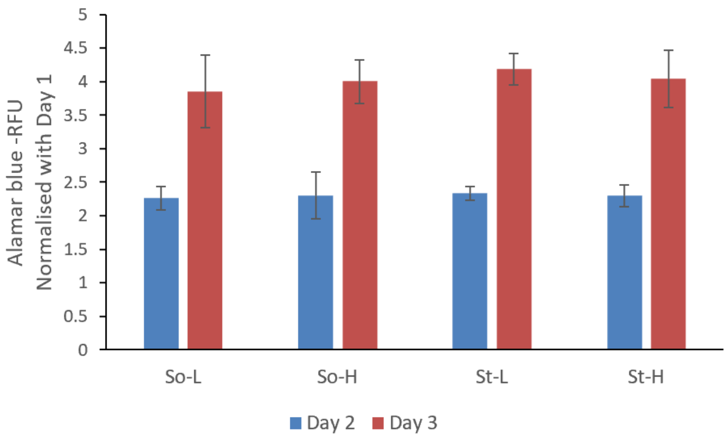

Figure S1. Scratch assay: Proliferation of MDA-MB 231 cells on 2D/TCP pre-conditioned in either So-L, So-H, St-L or St-H hydrogels.

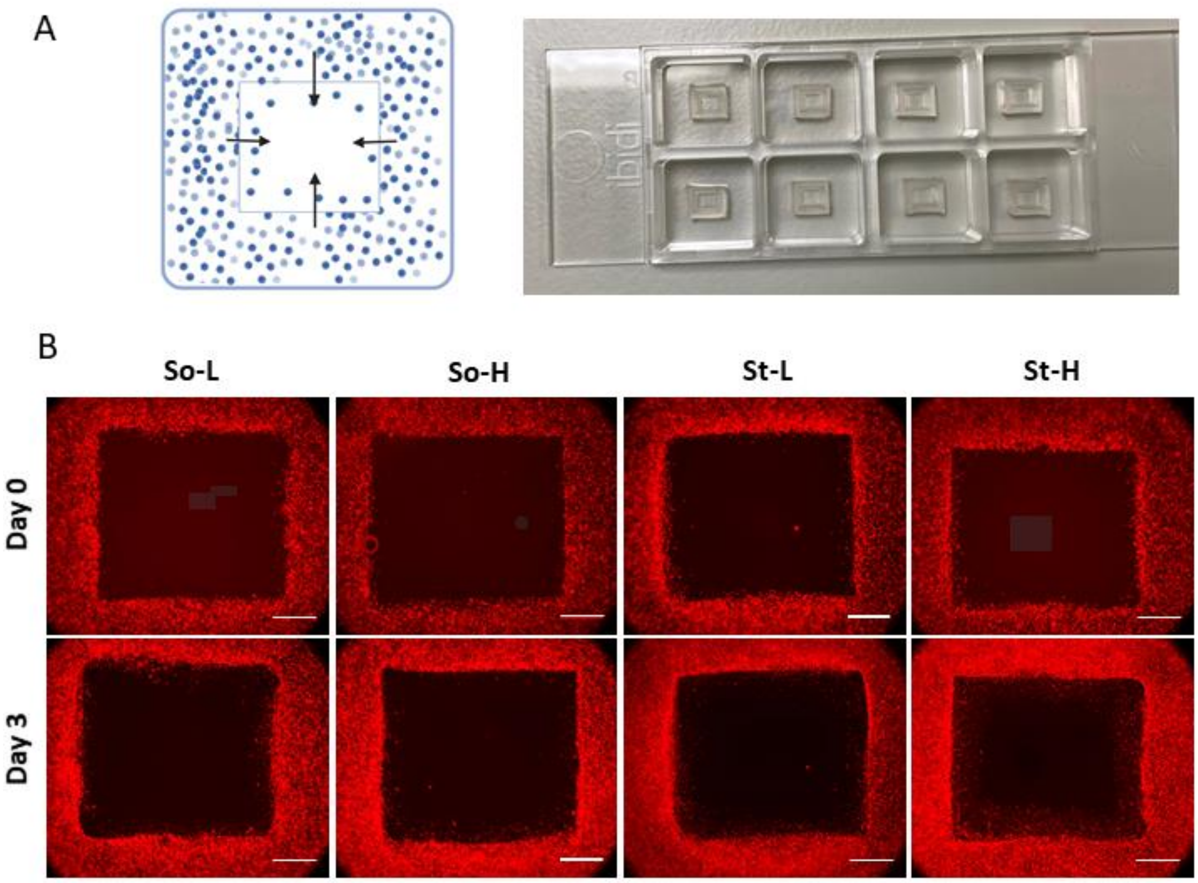

Figure S2. 3D Collagen invasion assay. (A) Schematic of invasion assay and representative image of inserts in 8-well imaging slides. **(B)** Cells stained with Cytopainter red were imaged at day 0 and day 3 of the assay (Scale bar- 1000 µm). Cells conditioned within four hydrogel groups of So-L, So-H, St-L and St-H were used in this assay. Insets and brightened image of day 3 invasion in acellular region showed in Figure 4.

SI.1 Alkaline Phosphatase assay (ALP)

At selected time points, cells were fixed with 4% v/v formaldehyde solution (1004968350, Sigma-Aldrich UK) for 10 min followed by washes with 1× PBS. The cells were then permeabilised with a solution of 0.1% v/v Triton-X in 1× PBS for 15 min and finally washed three times with 1× PBS. Cellular alkaline phosphatase activity (ALP) was quantified for each type of scaffold tested (n = 3) using ALP Diethanolamine activity kit (AP0100, Sigma-Aldrich, UK) following manufacturer's instructions. Briefly, after permeabilization, a 1 mL volume of ALP reaction buffer was added to the scaffolds. A 0.67 M pNPP substrate solution was prepared in ultrapure water and a 1 µL volume of this solution was added to the scaffolds, previously immersed in ALP buffer. Scaffolds were incubated for 15 min at 37 °C. The enzymatic activity was immediately measured with absorbance readings at 405 nm (Synergy-2 plat reader, Biotek, UK). Readings were converted to units/mL using a calibration curve obtained by measuring known amounts of ALP enzyme (U/mL) using the same method previously described and in the range of 0.15 U/mL and 10 U/mL. Finally, ALP activity was

normalised to cell number, with each U/mL values divided by the cell proliferation reading (Alamar blue) measured for the corresponding scaffolds. For each scaffold and time point, data are presented as mean ± SD (n = 2, N = 3).

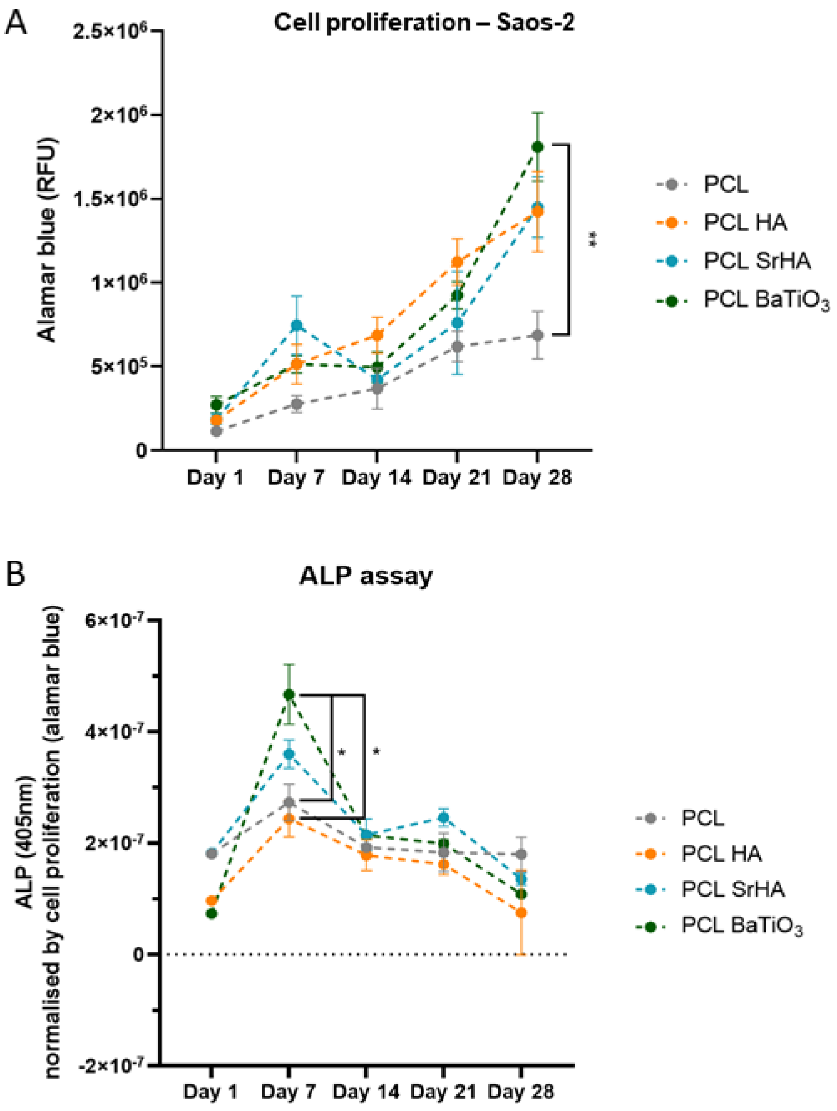

Figure S3. A) Saos-2 proliferation as measured by Alamar blue assay and **B)** ALP activity measured in Saos-2 cells when cultured in composite PCL scaffolds- PCL, PCL/HA, PCL/SrHA and PCL/$BaTiO_3$. Data is plotted as a line graph from day 1 to day 28 with a 7-day interval (average and SD of three independent experiments. P-values represented as *$p \leq 0.05$, **$p \leq 0.01$, ***$p \leq 0.001$, ****$p \leq 0.0001$

SI.2 Alizarin stain analysis

Calcium deposition was quantified using the Alizarin red stain. Briefly, scaffolds were fixed with 4% v/v formaldehyde solution at selected time points (day 21, day 28, n = 2, N = 3) or after decellularisation. After fixation, scaffolds were washed with ultrapure water and then incubated with Alizarin-red staining solution (TMS-008-C, Sigma-Aldrich, UK) for 15 min at

room temperature on a plate shaker. Excess stain was removed by additional washes (n = 3) with ultrapure water, then scaffolds were washed with acetone (n = 1) and left to dry at room temperature. To quantify the reacted and deposited alizarin stain on the scaffolds, a 2 mL volume of 0.2 M NaOH:MeOH (1:1) solution was added to dissolve the stain for each scaffold, then a volume of 200 µL was transferred to another well-plate and measured by absorbance reading at 405 nm (Synergy-2 plate reader, Biotek, UK). A calibration curve obtained from known concentration of Alizarin red stain (mM) in 0.2 M NaOH:MeOH was used to calculate the deposited calcium. For each scaffold and time point, data are presented as mean ± SD (n = 2, N = 3).

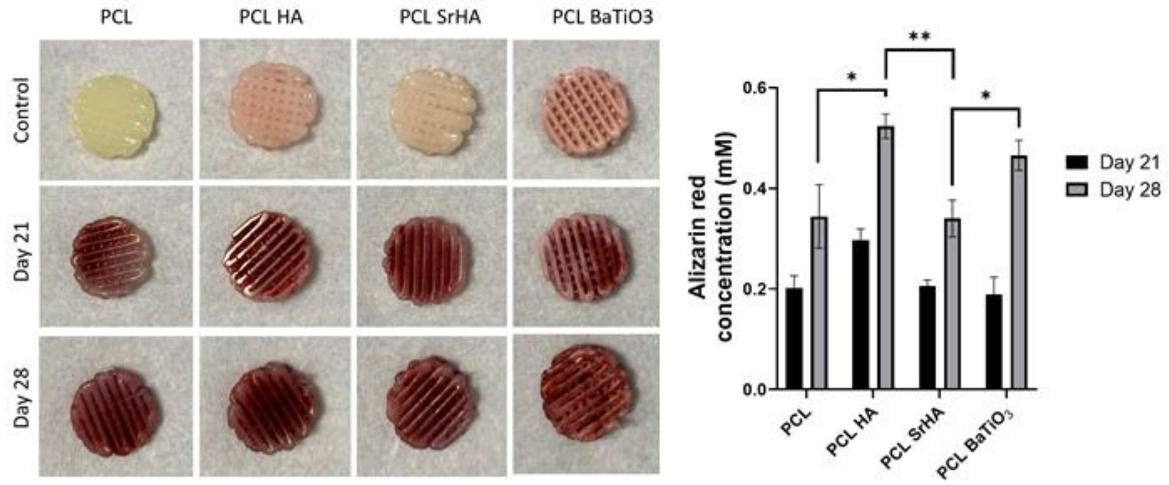

Figure S4. Calcium deposition analysis. PCL composite scaffolds stained with Alizarin stain on day 21 and day 28 of culture with Saos-2 cells (left). Control consisted of staining scaffolds with no cells cultured. Quantification of Alizarin stain on day 21 and day 28 and data represented as average and SD of three independent experiments (right). P-values represented as *p ≤ 0.05, **p ≤ 0.01, ***p ≤ 0.001, ****p ≤ 0.0001

SI.3 Collagen I and osteocalcin immunofluorescence (IF) staining

Staining was performed on the scaffolds to detect any deposition of extracellular matrix after 28 days of culture and after decellularization of scaffolds. For this staining, scaffolds were fixed with 4% v/v paraformaldehyde for 5 min at RT, washed three times with 1× PBS and then incubated with blocking buffer (1% w/v BSA in 1× PBS) for 1 h at RT to avoid non-specific antibody binding. Scaffolds were washed with 1× PBS, and then incubated with Osteocalcin monoclonal antibody (1:500 dilution in 1× PBS, MA1-82975, Thermofisher, UK) and Collagen-I polyclonal antibody (1:250 dilution in 1× PBS, PA5-95137, Thermofisher, UK) overnight (16 h) at 4 °C. After three washes with blocking buffer, samples were incubated with a solution of secondary antibodies Goat anti-rabbit 488 (1:2000 dilution in 1× PBS, A-11008, Thermofisher, UK) and Goat anti-mouse 594 (1:2000 dilution in 1× PBS, A-

11005, Thermofisher, UK) for 30 min at RT following manufacturers instruction. Samples were washed three times with blocking buffer and stored immersed in 1× PBS at 4 °C in the dark.

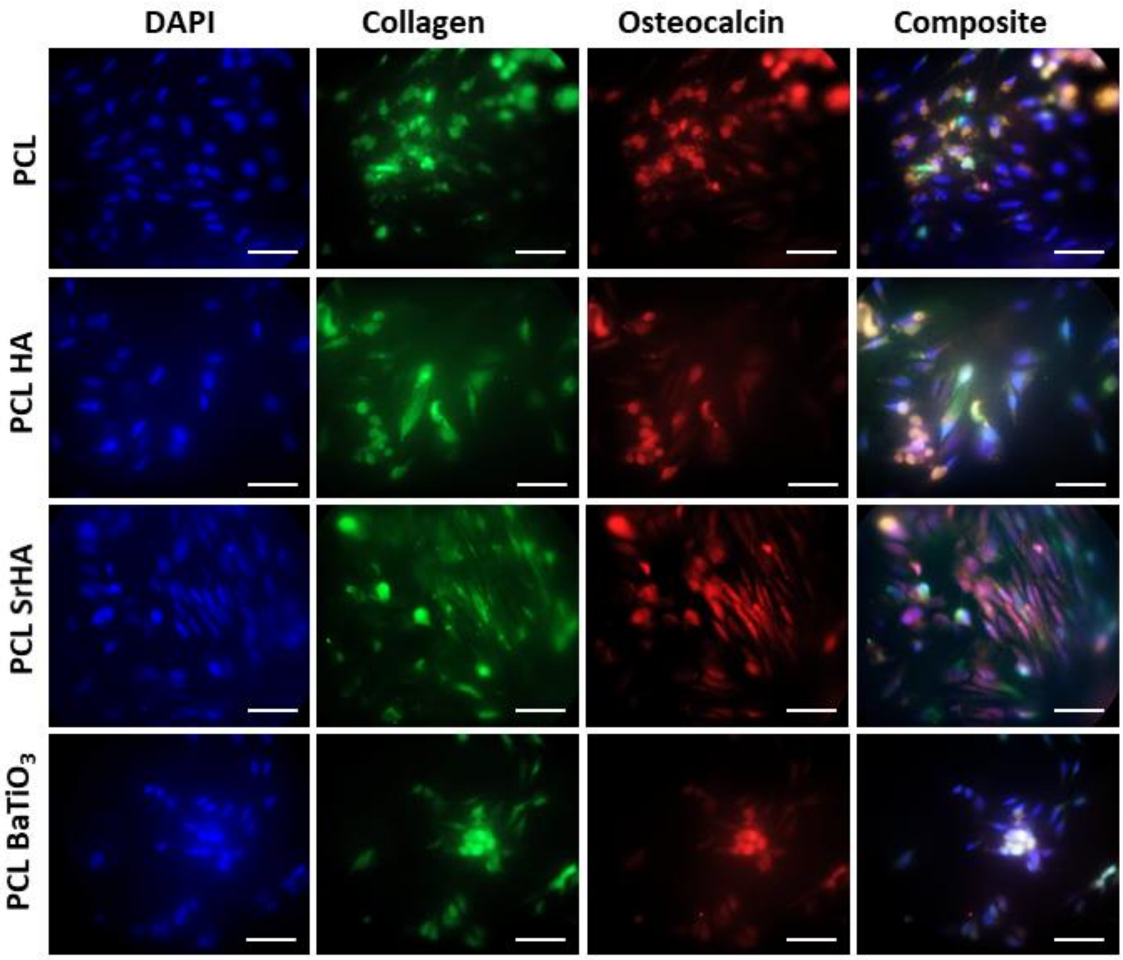

Figure S5. Collagen and osteocalcin deposition. Saos-2 cells were stained with DAPI, Collagen Ab and osteocalcin Ab on day 28 of culture in composite PCL scaffolds (Scale bars 50 µm). The presence of punctate staining near nucleus of cells is considered to be ECM deposited by cells.

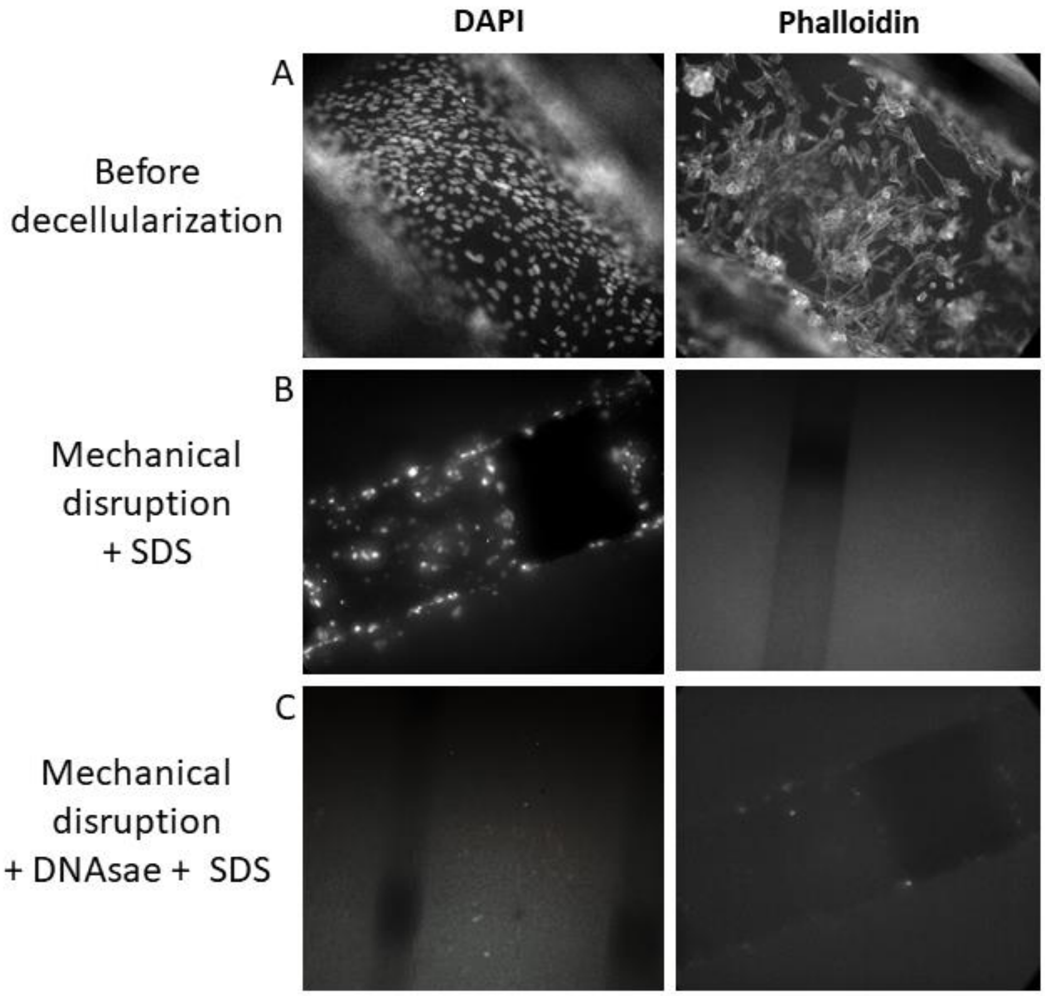

Figure S6. Standardisation of decellularization process in composite PCL scaffolds. DAPI and Phalloidin stained PCL scaffolds **A)** before decellularization, **B)** after mechanical lysis of cells and 0.05% SDS wash, and **C)** after mechanical lysis with SDS and 1mg/mL DNAase.

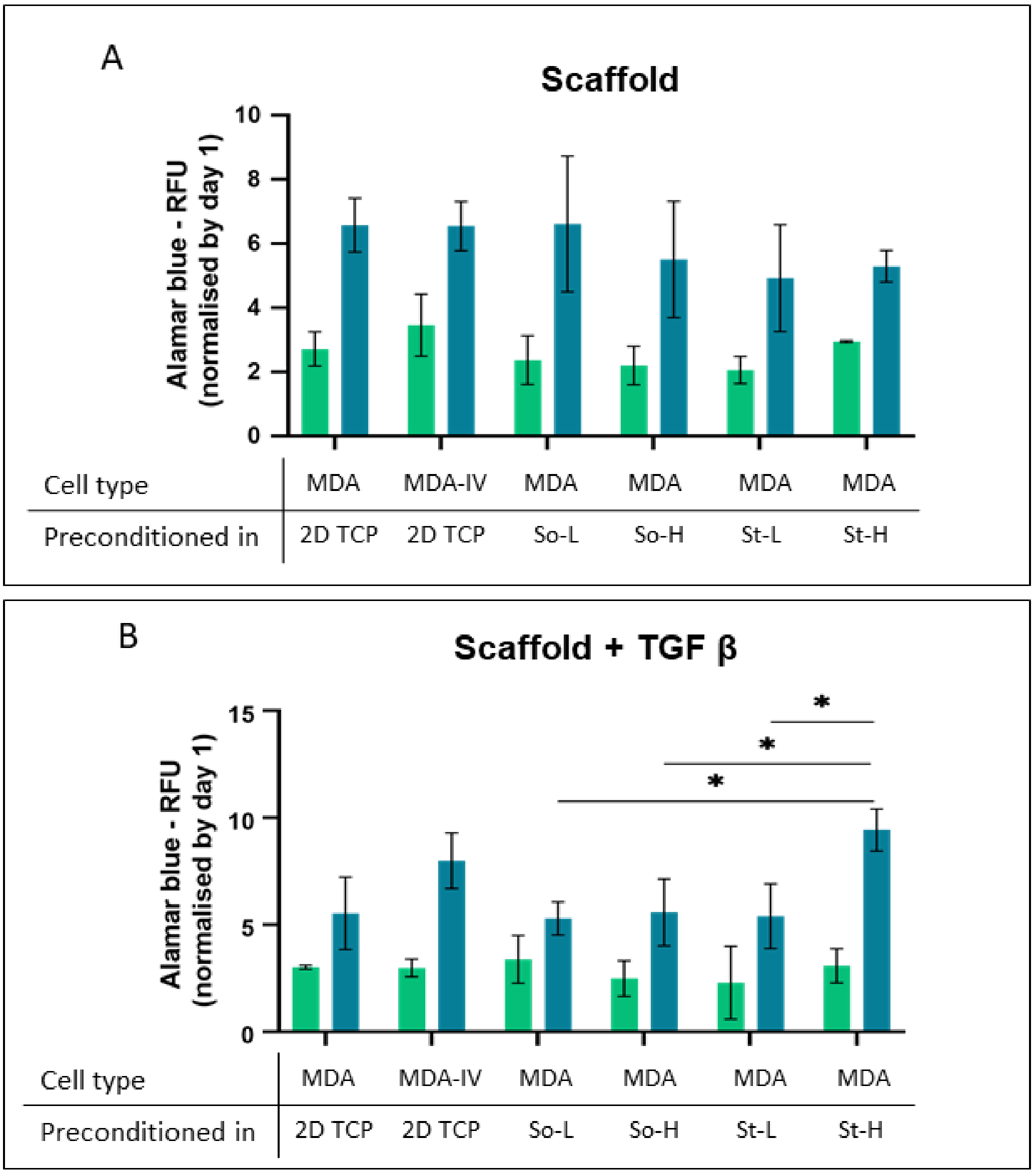

Figure S7. ***Indirect migration model*** **- Cell proliferation of breast cancer cells in biohybrid PCL scaffolds.** Alamar blue assay readings on day day 3 (green) and day 7 (blue) of MDA-MB 231 (represented as 'MDA') and MDA-IV cells pre-conditioned in either 2D TCP plates or alginate-gelatin hydrogels and plated on **A)** Scaffolds and **B)** Scaffolds + 5 ng/mL TGF-β1. Data is represented as average and SD of three independent experiments (right). P-values represented as $*p \leq 0.05$.

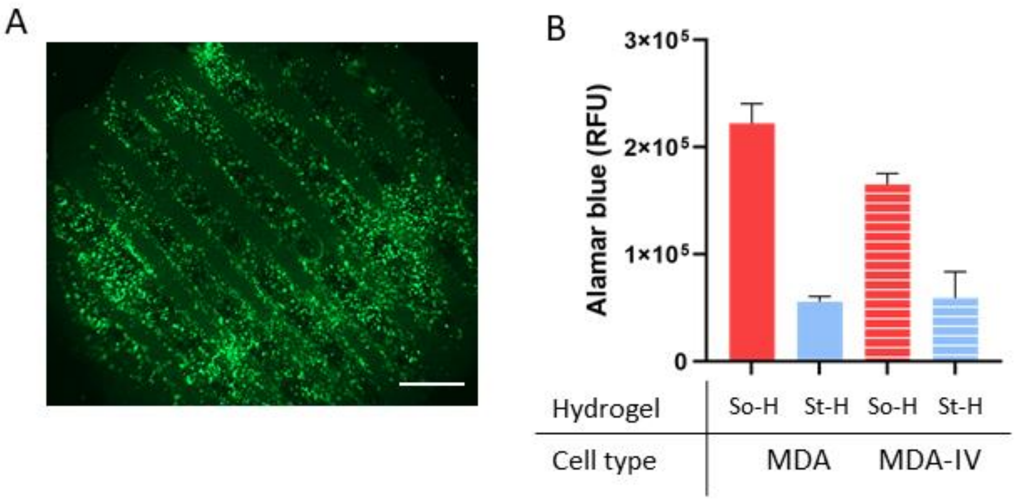

Figure S8. Migration quantified in *direct migration model*. A) Cells migrated from alginate hydrogels to biohybrid scaffolds stained with green Live stain (ethylene homodimer) at day 7 (Scale bar- 1000 µm). **B)** Quantification of migrated cells (MDA or MDA-IV) from So-H or St-H hydrogels to biohybrid PCL scaffold with Alamar blue assay at Day 7.
